# Fusing Residual and Cascade Attention Mechanisms in Voxel–RCNN for 3D Object Detection

**DOI:** 10.3390/s25175497

**Published:** 2025-09-04

**Authors:** You Lu, Yuwei Zhang, Xiangsuo Fan, Dengsheng Cai, Rui Gong

**Affiliations:** 1 School of Automation, Guangxi University of Science and Technology, Liuzhou 545000, China; 20230203009@stdmail.gxust.edu.cn (Y.L.); 100002085@gxust.edu.cn (X.F.); 20230201003@stdmail.gxust.edu.cn (R.G.); 2 Liugong Machinery Co., Ltd., Liuzhou 545000, China; caids@liugong.com

**Keywords:** residual connection, cascaded attention network, attention mechanism, 3D object detection

## Abstract

In this paper, a high-precision 3D object detector—Voxel–RCNN—is adopted as the baseline detector, and an improved detector named RCAVoxel-RCNN is proposed. To address various issues present in current mainstream 3D point cloud voxelisation methods, such as the suboptimal performance of Region Proposal Networks (RPNs) in generating candidate regions and the inadequate detection of small-scale objects caused by overly deep convolutional layers in both 3D and 2D backbone networks, this paper proposes a Cascade Attention Network (CAN). The CAN is designed to progressively refine and enhance the proposed regions, thereby producing more accurate detection results. Furthermore, a 3D Residual Network is introduced, which improves the representation of small objects by reducing the number of convolutional layers while incorporating residual connections. In the Bird’s-Eye View (BEV) feature extraction network, a Residual Attention Network (RAN) is developed. This follows a similar approach to the aforementioned 3D backbone network, leveraging the spatial awareness capabilities of the BEV. Additionally, the Squeeze-and-Excitation (SE) attention mechanism is incorporated to assign dynamic weights to features, allowing the network to focus more effectively on informative features. Experimental results on the KITTI validation dataset demonstrate the effectiveness of the proposed method, with detection accuracy for cars, pedestrians, and bicycles improving by 3.34%, 10.75%, and 4.61%, respectively, under the KITTI hard level. The primary evaluation metric adopted is the 3D Average Precision (AP), computed over 40 recall positions (R40). The Intersection over IoU thresholds used are 0.7 for cars and 0.5 for both pedestrians and bicycles.

## 1. Introduction

In recent years, with the rapid advancement of autonomous driving technologies, LiDAR sensors have become an essential component in the development of self-driving vehicles. The point cloud data they produce contain rich spatial information and offer reliable input for environmental perception, thereby attracting significant academic and industrial interest. Object detection is generally classified into two categories: two-dimensional (2D) and three-dimensional (3D). 3D object detection offers a more intuitive representation of the real world, incorporating size, orientation, and distance information [1]. As LiDAR technology matures and becomes more cost-effective, 3D point cloud-based object detection has emerged as a mainstream approach in autonomous driving. However, LiDAR-generated point clouds are unordered, uneven in density, and highly data-intensive, rendering direct application of 2D object detectors unsuitable for 3D data [2].

Research on point cloud-based object detection can be categorised into four main approaches according to the method of point cloud processing: point-based, voxel-based, projection-based, and hybrid point–voxel approaches. Point-based methods directly operate on raw point cloud data for feature extraction, effectively preserving geometric structures and spatial details. However, they are less suited to large-scale point cloud data. Representative works include PointNet, PointNet++, PointRCNN, and 3DSSD [3,4,5,6]. Voxel-based methods divide the point cloud into regular 3D voxel grids and model the spatial structure using voxel representations, which facilitates the extraction of local features in 3D space. The choice of voxel size significantly affects detection accuracy. Notable methods in this category include SECOND, VoxelNet, and Voxel–RCNN [7,8,9]. Projection-based approaches convert 3D point clouds into 2D images or pseudo-images, enabling the use of conventional 2D convolutional neural networks. This transformation simplifies the data while preserving the overall outline of objects. Representative methods include PointPillars and CenterPoint [10,11]. Hybrid point–voxel methods combine point-wise and voxel-wise representations to leverage both the fine-grained features of point clouds and the global structural information offered by voxels. These methods demonstrate strong object recognition capabilities across a variety of scenarios. Representative works include PV-RCNN, SASSD, and CT3D [12,13,14].

The baseline adopted in this study, Voxel–RCNN, is a classic and effective two-stage detector that not only extracts voxel-based features but also integrates BEV features [9]. Furthermore, the spatial awareness advantage provided by the BEV perspective enables the detector to identify smaller objects more effectively. Region proposals are first generated through the RPN, followed by refinement and classification to produce the final detection results. However, considering the limitations of this approach—namely, the suboptimal performance of the RPN and the excessive depth of both the 3D and 2D backbone networks—this paper proposes a CAN to iteratively refine the predictions. Additionally, residual connections are incorporated into both the 3D and 2D backbone networks. The 2D backbone further integrates a Squeeze-and-Excitation (SE) self-attention mechanism to dynamically reweight features, thereby enhancing the detection capability for small-scale objects [15].

Extensive experiments and rigorous ablation studies conducted on the KITTI dataset demonstrate the effectiveness of the proposed method. Specifically, the contributions of this work can be summarised as follows:

(1) To address the limited performance of the RPN, a Cascaded Attention Network is designed to iteratively refine region proposals and produce high-quality predictions.
(2) To mitigate the degradation in feature representation caused by excessively deep convolutional layers in both 3D and 2D backbones, a 3D Residual Network is introduced to enhance feature transmission across layers. Furthermore, a 2D Residual Attention Network is proposed to improve the model’s sensitivity to small-scale objects by incorporating attention-based feature weighting.
(3) The proposed method is validated on the KITTI dataset, with the results demonstrating its effectiveness. Under the hard setting of the 3D Average Precision (AP) metric using R40 evaluation, detection performance for cars, pedestrians, and cyclists improves by 3.34%, 10.75%, and 4.61%, respectively.

## 2. Related Work

Currently, 3D object detection methods can be broadly categorised into single-stage and two-stage detectors. The baseline adopted in this study is Voxel–RCNN, a two-stage detector proposed by Jiajun Deng et al. In the first stage, the point cloud is divided into a fixed-size voxel grid, and the point-wise information within each voxel is encoded to form voxel-wise feature representations. These features are then processed by a 3D convolutional backbone network to extract high-level voxel features, and the final-layer features are compressed along the height axis to generate a BEV representation [16]. Subsequently, a RPN applies a 2D backbone network to the BEV features for further extraction, and uses an anchor-based approach to generate candidate object regions [17]. In the second stage, in order to refine the proposals, Voxel–RCNN employs a Multilayer Perceptron (MLP) to directly extract fine-grained geometric features from the raw point cloud within each candidate region [9]. Finally, the Detection Head further refines these proposals and performs classification and 3D bounding box regression to produce the final detection results.

For single-stage detectors, object categories and bounding boxes are typically predicted directly from point cloud data. These methods feature a streamlined architecture that facilitates end-to-end modelling. PointNet and PointNet++, inspired by 2D object detection techniques, operate directly on raw point clouds, thereby effectively preserving geometric details effectively [3,4]. VoxelNet introduces a voxelisation approach, dividing the point cloud into fixed-size 3D grids and employing a Voxel Feature Encoding (VFE) module to extract voxel-level features, enabling an end-to-end learning framework from raw data to detection output [8]. Building upon voxelisation, SECOND addresses the sparse nature of point clouds by incorporating sparse 3D convolutions and applying max pooling to aggregate features, thereby enhancing representational capacity [7]. PointPillars partitions the point cloud into vertical columnar structures (pillars) and uses the Pillar Feature Net (PFN) to extract features from points within each pillar [10]. These features are then projected into a BEV representation and processed using 2D convolutional networks. This approach integrates the advantages of voxel partitioning and BEV representation in structural modelling. However, the construction of pillars may result in the loss of some fine-grained point cloud information, potentially affecting the accuracy of boundary detection. CenterPoint directly projects point clouds into the BEV space and extracts spatial features using 2D convolution, simplifying the processing pipeline while preserving the overall object shape [11]. Nevertheless, the flattening of the 3D structure inherent in BEV representations can lead to the weakening of certain spatial geometric cues. RangeDet projects the point cloud into a depth map based on a radar-view polar coordinate system, maintaining the original spatial structure of the data [18]. It leverages 2D convolutional networks for feature extraction while preserving spatial relationships. However, the geometric distortion and variation in object scales introduced by the range view projection can present challenges, particularly in detecting small or edge-located objects. DCGNN proposes a distinctive point set optimisation method based on density clustering and incorporates both local and global graph neural network modules, which are designed to model intra-set and inter-set relationships, respectively [19]. This approach facilitates a more comprehensive learning of spatial features and offers a novel perspective for single-stage detection networks.

Single-stage detectors lack a region proposal process, which limits their effectiveness in detecting smaller objects—especially when these objects are subject to geometric occlusion. Inspired by the Faster R-CNN architecture in 2D object detection, researchers have developed two-stage frameworks for 3D object detection [20]. In such frameworks, the first stage utilises an RPN to generate candidate regions from point cloud data, while the second stage performs refined feature extraction and bounding box regression on these proposals. This approach enhances detection accuracy, making it well-suited for high-precision 3D object detection tasks. PointRCNN directly extracts features from raw point clouds using PointNet++ and generates 3D object proposals. It then applies RoI pooling to further extract point-level features within each candidate region for object classification and bounding box refinement [5,21]. Although this method effectively leverages the raw geometric information of the point cloud and achieves high detection accuracy, its computational cost is relatively high, limiting its practicality in real-time applications. PV-RCNN processes point clouds through voxelisation and employs sparse voxel convolution to extract features and generate 3D object proposals [12]. It then further refines these proposals by fusing local voxel features with fine-grained raw point features, thereby improving detection accuracy [22]. However, the repeated computation of both voxel-based and point-based features leads to high computational overhead. To address this, PV-RCNN++ introduces improvements to the Voxel Set Abstraction (VSA) module [23], reducing unnecessary computation while maintaining high accuracy. Voxel Transformer combines the advantages of Transformer architectures and voxel-based representations [16], leveraging self-attention mechanisms for global feature extraction and thereby enhancing detection performance, particularly for distant objects. Point2Seq builds upon this by modelling object detection as a sequence generation problem [24]. It adopts an auto-regressive decoding scheme to progressively predict the bounding boxes, making it more effective for detecting small objects and handling sparse point clouds.

In summary, within the field of object detection, single-stage detectors are generally characterised by higher speed [25], whereas two-stage detectors often demonstrate superior accuracy, particularly for small objects. Direct processing of raw LiDAR point cloud data can yield high detection accuracy, but it inevitably poses significant computational challenges. Conversely, projecting point clouds into two-dimensional representations can reduce computational complexity, but often results in information loss and limits the ability to fully exploit the spatial structure of the data—especially when detecting small-scale objects [26]. The Voxel–RCNN algorithm combines voxel-based processing with well-established 2D convolutional techniques, effectively extracting both voxel-wise and BEV features [9]. However, as the depth of convolutional networks increases, the ability to detect small objects may deteriorate. To address this issue, the present study incorporates residual connections and the Squeeze-and-Excitation (SE) attention mechanism to enhance the network’s sensitivity to small objects. In addition, a CAN is introduced to enable multi-level refinement of detection results, thereby significantly improving overall detection accuracy. These improvements not only enhance the performance of the detection algorithm but also further unlock the potential of point cloud data in 3D object detection tasks.

## 3. Methods

**Figure 1:**
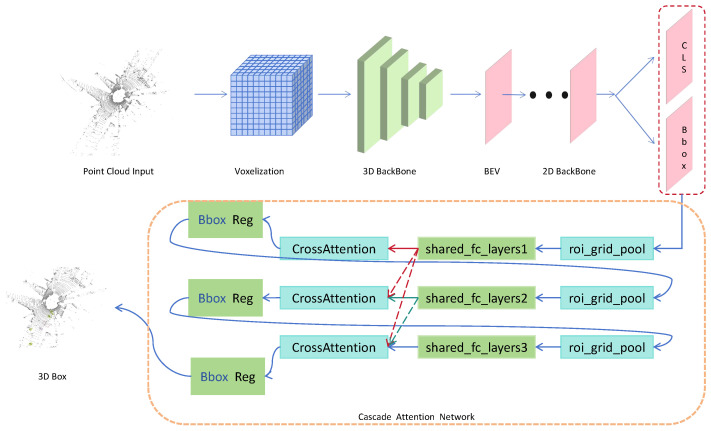
The overall framework diagram.

In this study, Voxel–RCNN is adopted as the baseline, with targeted enhancements introduced to address its existing limitations [9]. The proposed improvements primarily focus on three components: the 3D backbone network, the 2D backbone network, and the cascaded attention network applied after the RPN in the first stage. The overall framework of the proposed approach is illustrated in Figure 1.

### 3.1. Residual Backbone

**Figure 2:**
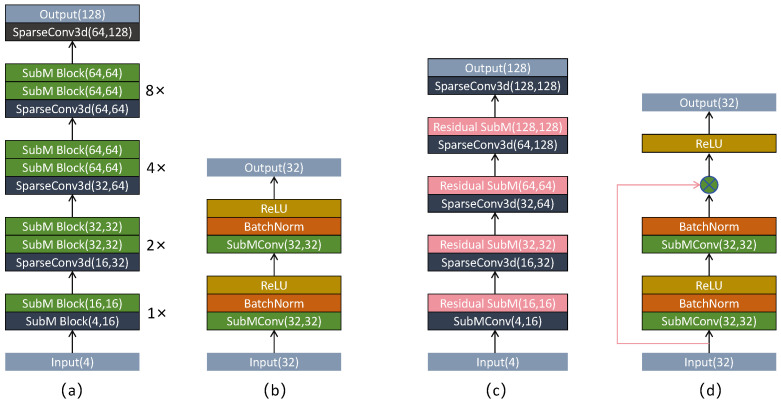
(**a**) Original network structure, (**b**) Subm block, (**c**) modified networkstructure, (**d**) Subm block with residual connections.

In Voxel–RCNN, the 3D backbone network is employed following voxel encoding [13], with the objective of extracting spatial features from non-empty voxels. This network primarily comprises Submanifold Convolution Blocks and Sparse Convolutions, which substantially reduce computational complexity while retaining rich feature representations for use in the subsequent RPN. As illustrated in Figure 2a, the 3D backbone network of Voxel–RCNN receives a four-dimensional voxel input containing the X, Y, and Z coordinates, as well as reflectance intensity. Initially, a Submanifold Convolution Block is applied to perform 1× downsampling [27], increasing the number of channels from 4 to 16. This is followed by an additional Submanifold Convolution Block for further feature extraction. Subsequently, sparse convolutions are employed to perform 2×, 4×, and 8× downsampling operations using a 3 × 3 × 3 kernel, with the number of channels gradually increased from 16 to 64 [28]. After each sparse convolution, two Submanifold Convolution Blocks are applied to extract features at multiple scales. The structure of a Submanifold Convolution Block is depicted in Figure 2b and consists of a submanifold convolution layer, normalisation layers, and activation functions. The original 3D backbone network adopts a linear structure, which means that feature extraction is carried out in a sequential manner. This approach results in insufficient multi-scale feature fusion, making it difficult for high-level abstract features to retain low-level detail information. Consequently, when features are subsequently compressed into the BEV representation, more low-level details are lost. To address this issue, residual connections have been integrated into the existing 3D backbone network. These connections facilitate the fusion and interaction of features across different levels, thereby enhancing the effectiveness of feature extraction in subsequent stages [29]. Specifically, the proposed method modifies the final two Submanifold Convolution Blocks, which are responsible for increasing the number of feature channels, by replacing them with residual Submanifold Convolution Blocks, as illustrated in Figure 2d. This design ensures that the features of small-scale objects are better preserved during the channel expansion process. Let *x* denote the original input, F(x) the output of the intermediate network layer, and *y* the output of the residual connection. The corresponding computation is defined as follows:

(1) $$\begin{array}{c} y=F(x)+x \end{array}$$

Meanwhile, during the 8× downsampling process, the number of input feature channels is maintained at 64, while the number of output feature channels is increased to 128 in order to extract more expressive features [30]. This enhancement facilitates spatial transformations and aggregation operations on the 3D feature map, enabling compression along the height dimension. As a result, the voxelised three-dimensional representation can be effectively projected onto a two-dimensional BEV. The modified network structure is illustrated in Figure 2c.

### 3.2. Residual Attention Network

**Figure 3:**
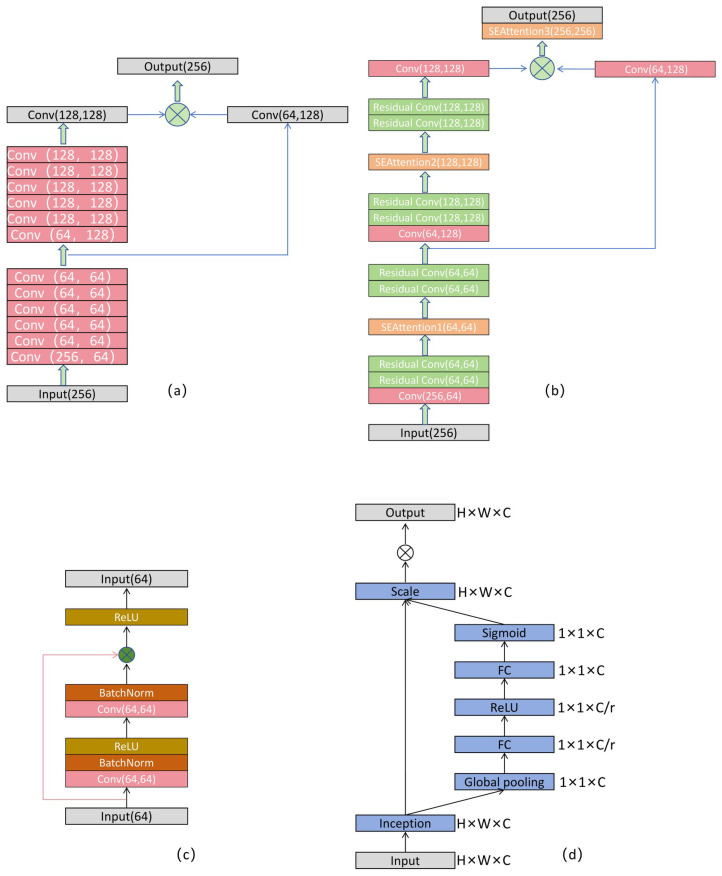
(**a**) Original 2D backbone network, (**b**) modified 2D backbone network, (**c**) residual convolutional block, (**d**) SE attention mechanism.

This study builds upon the final feature map extracted from the 3D backbone network, which is compressed along the height dimension (Z-axis) to generate a BEV feature map [31]. This transformation effectively simplifies the data structure, reduces computational complexity, and enhances the delineation of object boundaries, thereby benefiting subsequent feature extraction and region proposal generation. The existing 2D backbone network primarily comprises two stages—downsampling and upsampling—as illustrated in Figure 3a. Initially, during standard 2D CNN operations, the number of feature channels is reduced from 256 to 64, followed by five consecutive convolutional layers to extract deep features. Subsequently, a 2D convolutional layer is employed to perform 2× downsampling, while increasing the number of feature channels to 128. After downsampling, five additional convolutional layers are applied for further feature extraction. Finally, an upsampling convolution is used to restore the spatial resolution of the original feature map. To fully exploit multi-scale information, the number of feature channels is increased from 64 to 128 through a convolutional operation prior to downsampling, and the resulting features are fused with the upsampled feature map to produce a final fused feature map with 256 channels. However, during the feature extraction phase, the aforementioned framework suffers from a gradual weakening of feature representation for small-scale objects. This is primarily due to the sequential stacking of a large number of convolutional layers in the deeper stages of the network, which tends to dilute fine-grained information.

To address this issue, we propose an RAN. This network enhances the representation of key information by introducing residual connections and incorporating the SE attention mechanism [32]. The SE module suppresses redundant features and adaptively adjusts channel-wise feature weights, thereby improving the feature representation of small-scale objects. As a result, it provides higher-quality feature inputs to the RPN. As illustrated in Figure 3b. In this paper, a convolutional module based on residual learning is proposed, comprising two standard convolutional blocks arranged in series. Within this structure, an identity mapping of the input features is retained and combined with the output of the convolutional path via element-wise addition prior to the application of the activation function in the second convolutional block. This design, as illustrated in Figure 3c, facilitates direct information flow and enables efficient gradient propagation. The Squeeze-and-Excitation (SE) attention module employed in this work is capable of automatically learning the relative importance of feature channels, thereby enhancing the representational capacity of 3D point cloud features. The core idea is to increase the network’s sensitivity to salient features through a channel attention mechanism, while simultaneously suppressing redundant information. The squeeze phase compresses a feature map of dimensions W × H × C into a 1 × 1 × C feature vector by performing global average pooling across the spatial dimensions. This operation captures global contextual information and mitigates inter-channel dependency. Specifically, $z\in R^{C}$ is conducted by generating a statistic by reducing $U_{c}$ over the spatial dimensions H × W, with the following calculation formula:

(2) $$\begin{array}{cc} z_{c} & =F_{\mathrm{sq}}\left(u_{c}\right)=\frac{1}{W\times H}\sum_{i=1}^{W}\sum_{j=1}^{H}u_{c}\left(i,j\right) \end{array}$$

The Excitation stage employs an adaptive channel recalibration mechanism comprising two fully connected (FC) layers. The first FC layer reduces the number of channels from C to C/r, thereby decreasing computational overhead and introducing a non-linear transformation to enhance the feature representation capability. The second FC layer then restores the number of channels back to C and applies a Sigmoid activation function to generate normalised channel-wise attention weights. $W_{1}\in R^{\frac{C}{r}\times C},W_{2}\in R^{\frac{C}{r}\times C}$. The calculation formula is as follows:

(3) $$\begin{array}{cc} s & =F_{\mathrm{ex}}\left(z,W\right)=\sigma \left(g\left(z,W\right)\right)=\sigma \left(W_{2}\delta \left(W_{1}z\right)\right) \end{array}$$

Finally, the resulting channel attention weights are multiplied element-wise with the input features along the channel dimension, thereby adaptively modulating the representational capacity of each channel. The corresponding flowchart is presented in Figure 2d.

### 3.3. Cascade Attention Network

**Figure 4:**
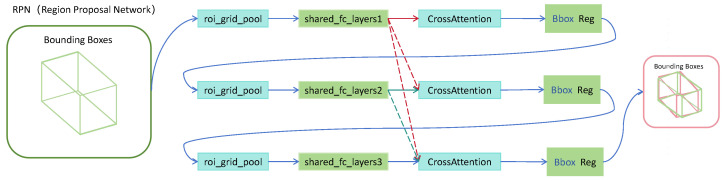
Cascade Attention Network structure.

Inspired by the remarkable performance of the 2D Cascade Attention Network in object detection tasks, this study extends its underlying principles to the domain of 3D object detection, and proposes a 3D Cascade Attention Network to improve detection accuracy and feature representation capability [33]. The overall process is illustrated in Figure 4. Initially, pooling operations are applied to the candidate regions generated by the RPN in order to extract features from within these regions. The primary aim of this step is to standardise Region of Interest (RoI) features of varying scales into a fixed size, thereby ensuring consistent input for subsequent processing via a Multi-Layer Perceptron (MLP). During the cascading phase, the features are iteratively refined and updated at each stage. To maintain the flow of global information and avoid redundant feature learning at individual stages, a Shared Feature Connection layer is introduced. Furthermore, to strengthen the network’s representational capacity, Cross Attention is incorporated into the cascade framework [34]. ${\hat{F}}^{j}$ denotes the encoded features from the previous stage, whereas $F^{j}$ represents the encoded features at the current stage. Here, $Q^{j}$, $K^{j}$ and $V^{j}$, corresponding to the query, key, and value, respectively. The computation is formulated as follows:

(4) $$\begin{array}{cc} {\hat{F}}_{i}^{j} & =\mathrm{softmax}\left(\frac{Q_{i}^{j}{\left(K_{i}^{j}\right)}^{T}}{\sqrt{c^{\prime }}}\right)V_{i}^{j} \end{array}$$

By learning the interactions between these features, the network is able to effectively integrate cross-stage information, thereby enhancing the robustness of object recognition. Ultimately, the optimised features are passed to the classification branch and regression branch, which are responsible for object category prediction and 3D bounding box regression, respectively, to produce the final detection results. The detailed process is illustrated in Figure 4.

### 3.4. Loss Function

The loss function in this article combines the RPN loss and the CAN loss with equal weights, $L=L_{RPN}+L_{CAN}$. The specific calculation formula is as follows:

(5) $$\begin{array}{cc} L_{\mathrm{RPN}} & =\frac{1}{N_{p}}\left[\sum_{i}L_{\mathrm{cls}}\left(a_{i},{\hat{a}}_{i}\right)+I\left(IoU_{i}>u\right)\sum_{i}L_{\mathrm{reg}}\left(\delta_{i},{\hat{\delta }}_{i}\right)\right] \end{array}$$

(6) $$\begin{array}{cc} L_{\mathrm{CAN}} & =\frac{1}{N_{b}}\left[\sum_{i}\sum_{j}L_{\mathrm{cls}}\left(a_{i}^{j},{\hat{a}}_{i}^{j}\right)+I\left(IoU_{i}^{j}>u^{j}\right)\sum_{i}\sum_{j}L_{\mathrm{reg}}\left(\delta_{i}^{j},{\hat{\delta }}_{i}^{j}\right)\right] \end{array}$$

For $L_{RPN}$, $N_{P}$ denotes the number of anchors. $a_{i}$ and $\delta_{i}$ represent the outputs for classification and bounding box regression, respectively, while ${\hat{a}}_{i}$ and ${\hat{\delta }}_{i}$ correspond to the classification labels and regression targets. L refers to the regions where the loss is computed, specifically those with an IoU greater than a predefined threshold. For the loss function of $L_{CAN}$, $a_{i}^{j}$ and $\delta_{i}^{j}$ represent the predicted results and ground truth targets, respectively, whereas ${\hat{a}}_{i}^{j}$ and ${\hat{\delta }}_{i}^{j}$ denote the predicted offsets and target offsets.

## 4. Experiment

### 4.1. Dataset and Evaluation Metrics

All model training and validation were conducted on the KITTI dataset, which consists of 7481 LiDAR point cloud samples for training and 7518 samples for testing. Following recent related works, the training set was further divided into 3712 samples for training and 3769 samples for validation. All network models were trained using the training split and evaluated on the validation split. For data augmentation, a mixed strategy was adopted. Specifically, random rotations and flipping were applied to prevent overfitting and to improve the generalisation capability of the models. The primary evaluation metric adopted is the 3D Average Precision (AP), computed over 40 recall positions (R40). The Intersection over IoU thresholds used are 0.7 for cars and 0.5 for both pedestrians and bicycles [35].

### 4.2. Experiment Details

All experiments were conducted on an NVIDIA GeForce GTX 3060 GPU with 12 GB of memory. The system was running Ubuntu 20.04, and the experiments were implemented using the PyTorch 1.10 deep learning framework, with CUDA 11.3 employed for model acceleration. The programming language used was Python 3.6.9. The point cloud input was restricted to the following spatial range: X-axis [0, 70.4] m, Y-axis [−40, 40] m, and Z-axis [−3, 1] m [35]. During the training phase, the number of training epochs was set to 80, and the batch size was fixed at 2. Other parameters, such as voxel size and initial learning rate, remained consistent with those provided in the publicly available code of the original paper.

### 4.3. Comparison with Other Algorithms

To evaluate the effectiveness of the proposed method, we conducted comparative experiments with several state-of-the-art algorithms on the KITTI validation dataset. The experimental results demonstrate that our approach achieves superior overall detection accuracy, with particularly notable improvements in the detection of small-scale objects. The detailed results are presented in Table 1.

### 4.4. Ablation Experiment

To thoroughly validate the effectiveness of the proposed approach, a series of systematic ablation studies were conducted to evaluate the individual contributions of each module to the overall model performance. Starting from the baseline model, the CAN, RAN, and 3D Residual module were introduced in succession. The experimental results demonstrate that the incorporation of these modules led to significant improvements in detection performance. Specifically, the 3D bounding box detection accuracy for cars, pedestrians, and bicycles under the three difficulty levels was improved by 0.47%, 3.09%, 3.34%, 10.71%, 11.81%, 10.75%, 5.61%, 4.67%, and 4.61%, respectively. The performance metrics for Voxel–RCNN were obtained using publicly available open-source code. Detailed results are provided in Table 2.

### 4.5. Visualisation of the Results

**Figure 5:**
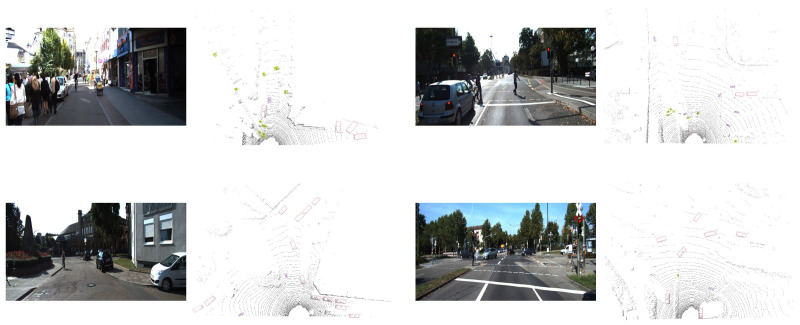
An example of results on the KITT validation dataset, featuring four different scenes, where cyclists, pedestrians, and bicycles are displayed in pink, green, and purple, respectively.

To provide a more intuitive understanding of the experimental results, visualisations were carried out using the KITTI dataset. During this process, raw point cloud data from multiple randomly selected scenes was input into the proposed model for inference. As illustrated in Figure 5, the 3D bounding box detection results across various scenes can be clearly observed, offering a visual representation of the model’s performance in diverse environments.

**Figure 6:**
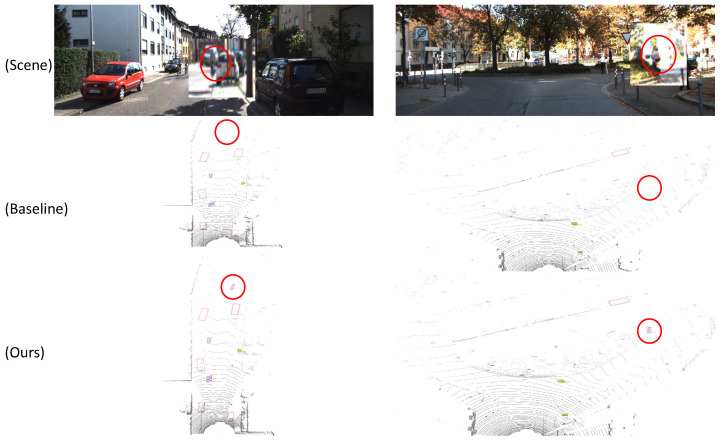
The detection results of distant targets in two different scenes, with the differences marked with circles.

The proposed model was compared with the baseline model across a range of scenarios. As illustrated in Figure 6, the model presented in this paper exhibits clear superiority in detecting small objects at long distances, thereby providing strong evidence of the effectiveness of the proposed modules.

**Figure 7:**
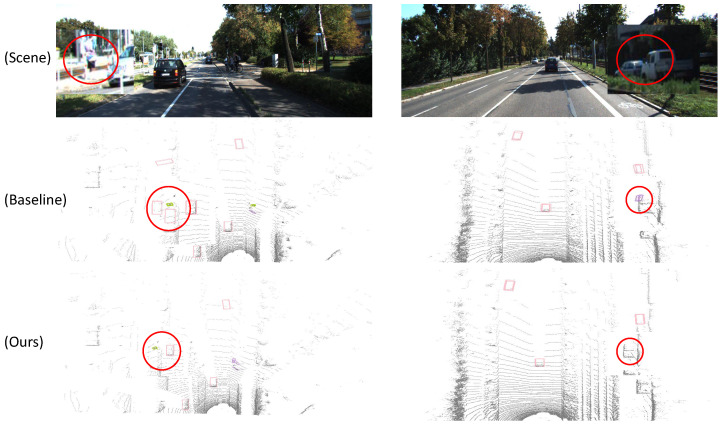
The detection results of distant targets in two different scenes, with the differences marked with circles.

Several scenarios were randomly selected for further comparison, and the results indicate that the model proposed in this study achieves a lower false detection rate compared to the baseline model. The detailed visual comparisons are presented in Figure 7.

## 5. Discussion

### 5.1. Limitations

Although the model proposed in this study has achieved notable improvements in overall detection accuracy, certain limitations remain. Firstly, the incorporation of the cascade attention network inevitably increases the computational complexity of the model. Secondly, in complex scenes, particularly where multiple small-scale objects are densely clustered, the model remains prone to missed detections.

### 5.2. Improvement Methods

In future research, we will focus on optimising the computational efficiency of the proposed algorithm. To address the issue of region redundancy in the three-stage region proposal mechanism of the CAN network, we plan to adopt a feature caching and reuse strategy [36]. Specifically, a feature memory bank will be constructed for overlapping regions, and during iterative processing, feature pooling operations will be performed only on newly generated regions. This approach is expected to significantly reduce computational complexity.

The issue of missed detections in scenarios involving dense clusters of small objects may be attributed to the use of overly large voxel sizes during the voxelisation process. In such cases, occluded small-object point clouds may become merged within a single voxel, making them difficult to distinguish. To address this, we intend to investigate the relationship between voxel size and detection accuracy in greater depth, with the aim of identifying an optimal voxelisation parameter configuration. Additionally, we plan to introduce a multi-scale feature fusion strategy to ensure that small objects across different scales receive adequate feature representation [37]. By enhancing the sensitivity and accuracy of the detection algorithm to small objects, this approach aims to improve the model’s capability in complex scenarios characterised by dense small-object distributions.

## 6. Summary

In this study, we designed three modules, namely, the CAN, RAN and 3D Residual modules, to enhance the detection performance of the model. The Cascade Attention Network (CAN) aggregates features from different stages using a cross-attention mechanism, which progressively improves and refines the region proposals generated by the Region Proposal Network (RPN). To address the problem of feature degradation caused by overly deep convolutional layers in the original 3D and 2D backbone networks, we reduced the number of convolutional layers and introduced residual connections. This design facilitates the transmission of information across different levels and improves the capability of the network to represent features effectively. The 3D Residual module successfully mitigates the limitations in the 3D backbone network. However, it is not suitable for the 2D backbone network used in the BEV representation, since the BEV is produced by compressing 3D feature maps along the height axis. This compression leads to weaker features for small objects. To resolve this issue, we developed the RAN, which incorporates the SE attention mechanism into the residual connection process. This mechanism adaptively adjusts the weights of different channels and enhances the feature representation of small-scale targets. Experimental results confirm that the algorithm proposed in this study achieves a clear improvement in overall detection accuracy, particularly in the detection of small objects.

## Figures and Tables

**Table 1:** In the comparison of algorithms, the best results are indicated in bold.

Methods	Car.3D (APR40)			Ped.3D (APR40)			Cyc.3D (APR40)		
	Easy	Mod	Hard	Easy	Mod	Hard	Easy	Mod	Hard
PV-RCNN (CVPR)	92.57	84.83	82.69	64.26	56.67	51.91	88.65	71.95	66.78
PV-RCNN++	92.11	85.55	82.27	67.74	60.55	55.92	88.73	73.58	69.05
PointPillars (CVPR)	86.42	77.29	75.60	53.6	48.36	45.22	82.83	64.24	60.05
SECOND (SENSOR)	90.55	81.86	78.61	55.94	51.14	46.17	82.96	66.74	65.34
CT3D (ICCV)	**92.85**	**85.82**	82.86	65.73	58.56	53.04	91.99	71.6	67.34
VoxelNet (CVPR)	87.88	75.58	72.77	56.46	50.97	45.65	78.18	61.74	54.68
$Part\text{-}A^{2}$ (TPAMI)	89.36	80.23	78.88	65.7	61.32	55.4	85.6	69.24	65.63
PointRCNN (CVPR)	89.8	78.65	78.05	62.69	55.77	52.65	84.48	66.37	60.83
**Ours**	92.81	85.78	**83.51**	**71.46**	**64.74**	**58.72**	**92.92**	**76.61**	**71.62**
	−0.04	−0.04	+0.65	+5.74	+3.42	+3.32	+0.93	+4.66	+4.28

**Table 2:** Improvement in performance through different modules, the best results of this paper’s algorithm are indicated in bold.

Method	Module				Car.3D (APR40)			Ped (APR40)			Cyc.3D (APR40)		
	CAN	RAN		3D Residual	Easy	Mod	Hard	Easy	Mod	Hard	Easy	Mod	Hard
		RN	RAN										
Voxel–RCNN					92.34	82.71	80.17	60.75	52.93	47.97	87.31	71.94	67.43
A	✓				92.47	83.85	82.18	69.80	61.53	56.88	90.82	73.53	68.88
B	✓	✓			92.58	83.86	82.33	69.9	61.54	56.95	91.22	73.46	69.91
C	✓		✓		92.80	84.93	82.99	70.13	62.52	57.31	91.85	74.05	69.92
**Ours**	✓		✓	✓	**92.81**	**85.78**	**83.51**	**71.46**	**64.74**	**58.72**	**92.92**	**76.61**	**71.62**
Improvement					+0.47	+3.09	+3.34	+10.71	+11.81	+10.75	+5.61	+4.67	+4.61

## Data Availability

The raw data supporting the conclusions of this article will be made available by the authors upon request.

## Abbreviations

The following abbreviations are used in this manuscript:

RCAVoxel-RCNN	Residual and Cascade Attention Mechanisms in Voxel–RCNN
RPN	Region Proposal Network
CAN	Cascade Attention Network
RAN	Residual Attention Network
SE	Squeeze-and-Excitation
BEV	Bird’s-Eye View
3D	Three-Dimensional
2D	Two-Dimensional
VFE	Voxel Feature Encoding
PFN	Pillar Feature Net PFN
VSA	Voxel Set Abstraction
FC	Fully Connected
